# The retinoic acid binding protein CRABP2 is increased in murine models of degenerative joint disease

**DOI:** 10.1186/ar2604

**Published:** 2009-01-28

**Authors:** Ian D Welch, Matthew F Cowan, Frank Beier, Tully M Underhill

**Affiliations:** 1 Department of Animal Care and Veterinary Services, University of Western Ontario, London, Ontario, N6A 5C1, Canada; 2 Department of Cellular and Physiological Sciences, The University of British Columbia, Vancouver, British Columbia, V6T 1Z3, Canada; 3 Department of Physiology and Pharmacology, CIHR Group in Skeletal Development and Remodeling, The University of Western Ontario, London, Ontario, N6A 5C1, Canada

## Abstract

**Introduction:**

Osteoarthritis (OA) is a debilitating disease with poorly defined aetiology. Multiple signals are involved in directing the formation of cartilage during development and the vitamin A derivatives, the retinoids, figure prominently in embryonic cartilage formation. In the present study, we examined the expression of a retinoid-regulated gene in murine models of OA.

**Methods:**

Mild and moderate forms of an OA-like degenerative disease were created in the mouse stifle joint by meniscotibial transection (MTX) and partial meniscectomy (PMX), respectively. Joint histopathology was scored using an Osteoarthritis Research Society International (OARSI) system and gene expression (*Col1a1*, *Col10a1*, *Sox9 *and *Crabp2*) in individual joints was determined using TaqMan quantitative PCR on RNA from microdissected articular knee cartilage.

**Results:**

For MTX, there was a significant increase in the joint score at 10 weeks (n = 4, p < 0.001) in comparison to sham surgeries. PMX surgery was slightly more severe and produced significant changes in joint score at six (n = 4, p < 0.01), eight (n = 4, p < 0.001) and 10 (n = 4, p < 0.001) weeks. The expression of *Col1a1 *was increased in both surgical models at two, four and six weeks post-surgery. In contrast, *Col10a1 *and *Sox9 *for the most part showed no significant difference in expression from two to six weeks post-surgery. *Crabp2 *expression is induced upon activation of the retinoid signalling pathway. At two weeks after surgery in the MTX and PMX animals, *Crabp2 *expression was increased about 18-fold and about 10-fold over the sham control, respectively. By 10 weeks, *Crabp2 *expression was increased about three-fold (n = 7, not significant) in the MTX animals and about five-fold (n = 7, p < 0.05) in the PMX animals in comparison to the contralateral control joint.

**Conclusions:**

Together, these findings suggest that the retinoid signalling pathway is activated early in the osteoarthritic process and is sustained during the course of the disease.

## Introduction

Osteoarthritis (OA) is a degenerative joint disease (DJD) that impacts multiple joint tissues (i.e. subchondral bone, synovium), but is typically associated with a deterioration of articular cartilage. Although numerous factors have been suggested to be important contributors to the development and progression of this disease, very few, with the possible exception of *FRZB *or *GDF5*, have been confirmed to have causal roles [1]. OA is considered in many instances to result from years of wear and tear on the joint. In this scenario, as with many other structures and organs within the body, the cartilage is considered to wear out as a result of ageing. Therefore, OA usually develops over a protracted period, which can be accelerated in certain individuals because of an underlying genetic predisposition or various environmental factors.

In the past few years, genetic links to OA have been established, and the first mutations in the collagen type II gene involved in the disease described [2,3]. More recently, other genes associated with the WNT and GDF signalling pathways have been implicated in OA susceptibility [4-6]. With regard to environmental factors, the biggest contributor is most likely to be physical activity/trauma and underlying medical conditions that place a greater mechanical burden on articular cartilage. The net result of these various factors is the loss of the integrity of the cartilaginous extracellular matrix (ECM), leading to a decrease in mechanical strength. This increases the susceptibility of the articular cartilage to further damage, and because of its limited ability to repair itself the disease worsens. In OA, the structural integrity of the matrix is irreversibly lost, leading to joint dysfunction [7].

One class of molecules that is important in development and homeostasis are the metabolites of vitamin A, the retinoids [8,9]. In the developing mammalian limb, retinoic acid has long been known to affect cells of mesenchymal and chondrogenic origin [10-13]. The addition of retinoic acid to high-density cultures of limb bud mesenchymal cells (which form cartilage nodules *in vitro*) has been shown to decrease the number and size of cartilage nodules formed. More interestingly, treatment of mature chondrocytes with retinoic acid causes them to assume an immature phenotype [14-17]. This is accompanied by a decrease in *Col2a1 *expression [14] and an increase in metalloproteinase expression [18] that leads to degradation of the ECM. In this regard, retinoic acid treatment of cartilage is commonly used to study cartilage degeneration [19,20]. *In vivo*, intra-articular injection of retinoic acid leads to chondrocyte dedifferentiation and DJD [21]. More recently, antagonists of the retinoic acid receptors have been tested in a rheumatoid arthritis model in mice and rats and found to improve histological scores, and this was associated with decreased expression of *Mmp13 *[22].

The changes in aggrecan metabolism seen in OA are similar to those produced by treatment of cartilage with retinoic acid. Bovine cartilage explant cultures treated with retinoic acid exhibit increased degradation of proteoglycans [23]. In rat osteosarcoma cells and primary bovine chondrocytes, treatment with retinoic acid produces cleavage of aggrecan (ACAN) at the E373-A374 peptide bond that is also cleaved in OA [24]. The retinoic acid-mediated degradation of ACAN is inhibited by metalloprotease inhibitors, but not by inhibitors of cathepsin B [23]. Others have shown that the addition of retinoic acid to chondrocytes stimulates maturation and hypertrophy consistent with the effects observed *in vivo *[25,26]. A switch from type II expression to type X and a decrease in ACAN expression accompanied by an increase in catabolism of collagen type II and ACAN was observed. In this regard, retinoic acid has been shown to enhance chondrocyte hypertrophy both *in vitro *and *in vivo*, where retinoic acid was observed to promote premature closure of the growth plate [27,28].

To examine the status of the retinoic acid signalling pathway in OA, we have used two murine DJD models and quantified the expression of a retinoic acid-regulated gene, including *Crabp2 *in the articular cartilage. In a recent study, *Crabp2 *was found to be elevated in DJD in a rat model of OA [29,30]. We found *Crapb2 *to be significantly increased in early OA, indicating that the retinoic acid pathway may play a role in OA pathophysiology.

## Materials and methods

### Surgery

Surgeries were performed on 10-week-old male C57BL/6NCr1 mice (Charles River Laboratories, St. Constant, Quebec, Canada). After a one-week period of acclimation after arrival, mice were sorted into random groups using a lottery system. For each experimental time point a minimum of five mice were evaluated. Mice were induced with 4% isoflurane and 1 L/minute oxygen. Once mice were anaesthetised they were transferred to a mask and maintained on 2% isoflurane and 0.8 L/minute oxygen. The surgical area was shaved and a three-part preparation was applied containing hibitane soap (Ayerst, Montreal, Quebec, Canada), isopropyl alcohol and betadine solution (Purdue Pharma, Pickering, Ontario, Canada).

**Figure 1:**
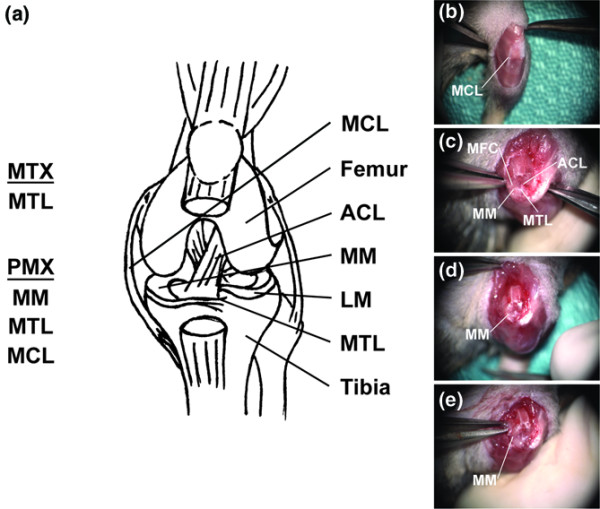
Overview of the surgical procedures used to generate meniscotibial transection (MTX) and partial meniscoectomy (PMX) models. **(a) **Schematic representation of the joint (Adapted from Kamekura and colleagues [34]). **(b) **An incision through the skin and subcutaneous tissue exposes the stifle joint. Prominent features include the medial collateral ligament (MCL), the patellar ligament and the tibial tuberosity. **(c) **The joint capsule has been cut on the medial side of the joint and the patella has been reflected laterally. This exposes the internal structures of the joint. Prominent features include the medial femoral chondyle (MFC), the medial meniscus (MM), the meniscotibial ligament (MTL) and the anterior cruciate ligament (ACL). **(d) **The MTL has been cut to release the MM. **(e) **The partial meniscectomy has been completed and the cut edge of the MM can be seen. Note that in order to visualise the cut edge of the meniscus, for pictorial purposes, the anterior cruciate ligament (ACL) has been transected. LM, lateral meniscus.

During surgery, the body temperature of the animals was maintained by placing them on a warm circulating water pad. Standard sterile techniques were used throughout the surgery. A small sterile drape was fitted over each mouse to expose only the area of interest, the medial side of the left knee. The surgery began with a small skin incision starting from the distal femur and extending to the proximal tibia on the medial side of the knee. The subcutaneous tissue was dissected throughout the length of the skin incision. The deep fascia that connects the parapatellar fascia to the biceps femoris was separated exposing the medial side of the joint including the medial collateral ligament (MCL) and the joint capsule. In the partial meniscectomy (PMX) surgical paradigm both the MCL and the joint capsule were incised to reveal the medial meniscus. The medial meniscus was gently held and retracted in a way that allowed for identification and transection of the meniscotibial ligament (Figure 1). The medial meniscus can subsequently be observed to be 'free' at its dorsal border. The joint capsule incision was continued medially progressing to the caudomedial edge of the tibial plateau. Gentle traction on the medial meniscus allowed the meniscus to be isolated and transected (Figure 1).

In the meniscotibial transection (MTX) surgical paradigm there was no medial dissection of the joint capsule or transection of the MCL. In this regard, the MTX model is similar to the destabilisation of the medial meniscus (DMM) model recently described by Glasson and colleagues [31]. Once the procedure was completed the deep fascia was closed using an interrupted suturing pattern with 5-0 vicryl (Ethicon Inc., Markham, Ontario, Canada). The subcutaneous tissue and skin were also closed together in a continuous subcuticular pattern using 5-0 vicryl. Any loose skin edges were apposed with sterile surgical glue. On completion of the surgery the inhalation anaesthesia was turned off and to control pain and infection, the mice were injected subcutaneously with buprenorphine (Schering-Plough, Hertfordshire, UK) and ampicillin (Novopharm, Toronto, Ontario, Canada) in normal physiological saline. The sham operation involved a similar incision to the left knee without compromising the joint capsule. All animal experiments were sanctioned by The University of Western Ontario's Animal Care Committee and conducted in full compliance with the Canadian Council on Animal Care.

### Joint histology

At the time of processing, mice were euthanased by carbon dioxide inhalation and both stifle joints were harvested and fixed in 4% paraformaldehyde. After a minimum of 48 hours fixation the joints were decalcified for 96 hours in 26% formic acid (TBD-2, Thermo Inc., Pittsburgh, PA, USA). Once decalcified the knees were paraffin embedded and serially sectioned in a sagital plane starting on the medial edge of the joint. Slides were stained with safranin-O and the medial side of tibial plateaus were subsequently scored according to Pritzker and colleagues from a minimum of three slides [32]. The section with the highest score was recorded. Briefly, this scoring system, ranging from 1 to 24, involves the product of the horizontal extent of the OA by the vertical severity of any lesions present.

### RNA isolation and quantitative PCR

At predetermined endpoints the mice were euthanased and the knees were carefully dissected to expose the cartilage surface of both tibial plateau and the femoral chondyles. Under an operating microscope, using a pair of micro-rongeurs, the articular cartilage was gently scraped away from the underlying subchondral bone and transferred into Qiazol (Qiagen, Mississauga, Ontario, Canada). The cartilage was subsequently homogenised in a microcentrifuge tube using a disposable plastic pestle and stored at -80°C. RNA was isolated from the samples according to the manufacturer's guidelines and for real-time quantitative PCR (RT-qPCR) the RNA was reverse-transcribed using the High Capacity cDNA Reverse Transcription Kit (Applied Biosystems, Foster City, CA, USA). Gene expression was quantitated using qPCR on an ABI 7500 Fast system with either custom TaqMan MGB probe or primer sets (*Col1a1, Sox9*) or off the shelf TaqMan Gene Expression Assays (Applied Biosystems, Foster City, CA, USA). Relative expression was determined using the relative quantitation method with a standard curve and gene expression was normalised to 18S abundance.

### Statistical analyses

For multiple comparisons, significance was determined using analysis of variance (ANOVA) with Bonferroni's or Tukey's post-hoc tests as indicated. With the exception of the analysis of *Crabp2 *in the 10-week-old mice, all tests in the gene expression studies were made between the operated left knee and the contralateral unoperated right knee and a sham. For comparison between operated and the contralateral knee in the 10-week-old mice significance was determined using two-tailed t-test. Significance is represented as follows: * p < 0.05; ** p < 0.01; *** p < 0.001.

## Results and discussion

In an earlier study of a rat surgically induced model of DJD, Appleton and colleagues reported that *Crabp2 *expression as determined from microarray analysis was elevated in the OA joint four weeks post-surgery [30]. *Crabp2 *is regulated by retinoic acid and is often induced upon activation of this pathway [33]. We were interested in confirming these findings and determining the kinetics of *Crabp2 *induction during OA. Further, we desired to develop and validate murine cartilage-sparing models of OA to facilitate the use of genetically modified lines and enable molecular analysis of gene expression in the articular cartilage. For these purposes, surgeries were tailored to produce less joint instability with the intent of producing a more slowly progressing disease than the standard anterior cruciate ligament (ACL) models as has been recently described for the DMM and other models [31,34].

**Figure 2:**
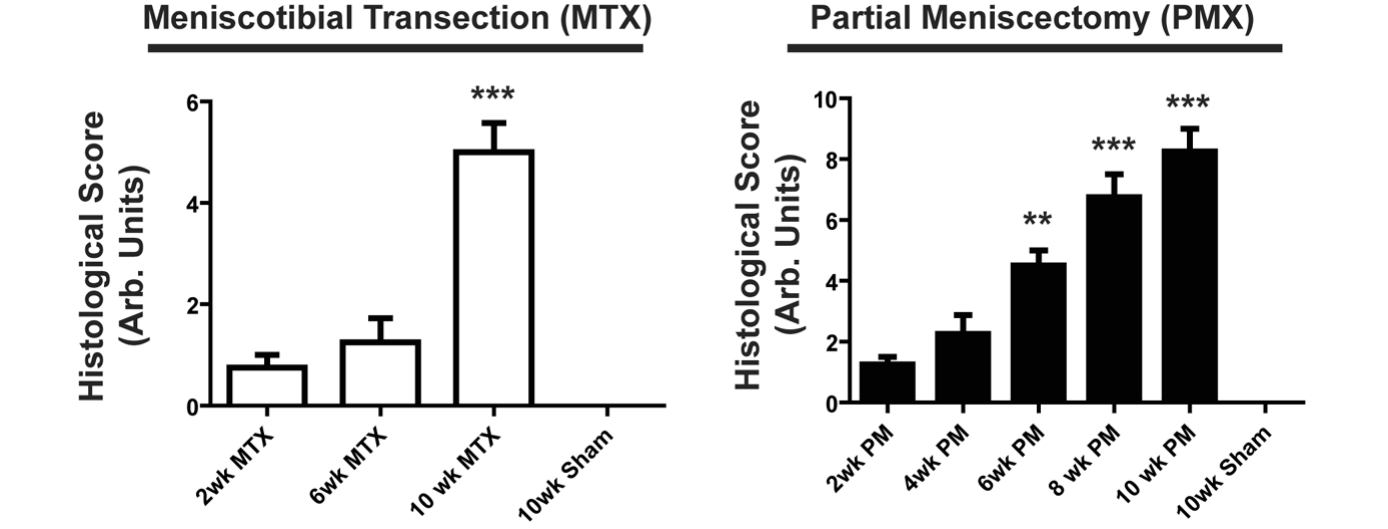
Meniscotibial transection (MTX) and partial meniscoectomy (PMX) surgeries lead to degenerative changes in the joint as evaluated by joint scoring. Joints were scored based on the Osteoarthritis Research Society International (OARSI) system. The PMX surgery was associated with a higher histological score than that of the MTX surgery. The sham surgeries at week 10 had a 0 score. ** p < 0.01; *** p < 0.001.

**Figure 3:**
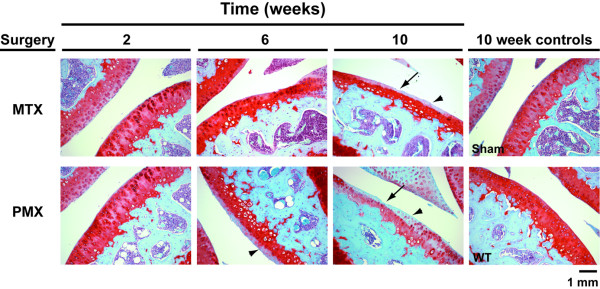
Meniscotibial transection (MTX) and partial meniscoectomy (PMX) surgeries lead to degenerative joint disease in mouse knees. Histological sections were collected at the indicated time points and stained with safranin O. Note in both the six-week and 10-week PMX and 10-week MTX surgery groups the loss of proteoglycan staining (arrowhead) in the superficial layers and in the 10-week PMX there is delamination of the superficial layer (arrow). WT, wild-type.

MTX and PMX surgeries were performed and joint histology was scored at various times up to 10 weeks post surgery. In the MTX group there was a significant increase in the joint score by 10 weeks (n = 7, p < 0.001), although increases at two and six weeks were not significant (Figure 2). In the recently reported DMM model, significant joint deterioration was observed at four weeks, and the differences between this model and the related MTX model may be a consequence of the different mouse strains used (129/SvEv versus the C57BL/6NCr1 strain utilised herein) and/or the different scoring methodologies employed [31]. Consistent with the more aggressive nature of the surgery, the PMX mice displayed a significant increase in joint score by six weeks after surgery (n = 7, p < 0.01); at 10 weeks these animals had a joint score of 8.4 (n = 5) out of a possible total of 24 (Figure 2). The early histopathological findings are focal, superficial to deep fibrillation sometimes associated with variable degrees of matrix depletion in these early stages. In the more aggressive PMX surgical paradigm the lesions become more diffuse and are more likely to have vertical fissures through the midzone with less common delamination of the superficial layer (Figure 3). Together, these results demonstrate that the PMX and MTX surgeries give rise to moderate and mild forms of DJD that are associated with a slowly progressing joint disease.

**Figure 4:**
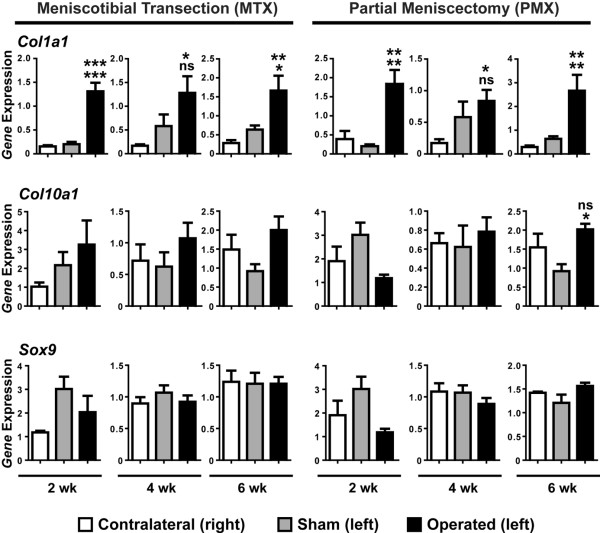
Analysis of *Col1a1*, *Col10a1 *and *Sox9 *expression in knee cartilage from meniscotibial transection (MTX), partial meniscoectomy (PMX) and sham surgeries. Genes analysed are shown on the left and significance was determined by analysis of variance (ANOVA) with Tukey's post-hoc tests for multiple comparisons. In comparison to *Sox9 *and *Col10a1*, *Col1a1 *is significantly increased at multiple time points in the different surgeries. Gene expression was normalised to an 18S internal control and the normalised expression (arbitrary units) for each gene is shown for the contralateral unoperated right knee, a sham control (left) knee (n = 5) and the operated left knee. Significance to the contralateral and sham controls is indicated on the top and bottom, respectively. * p < 0.05; ** p < 0.01; *** p < 0.001; ns = not significant.

**Figure 5:**
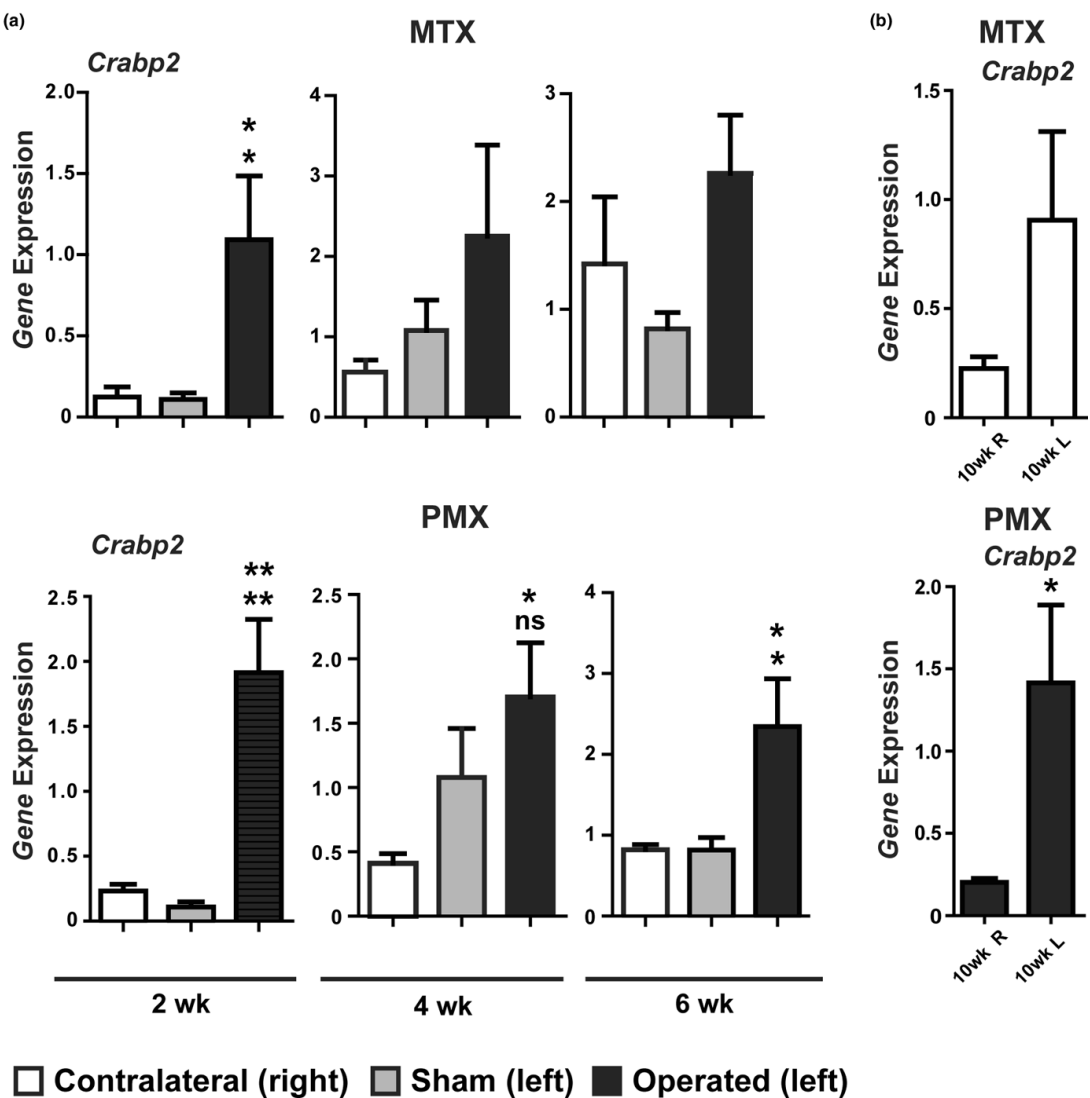
*Crabp2 *expression is increased in joint cartilage from meniscotibial transection (MTX) and partial meniscoectomy (PMX) operated knees. **(a) ***Crabp2 *expression was analysed at two, four and six weeks post-surgery and its expression was significantly changed in MTX/PMX-operated knees (n = 5 to 7) in comparison to either the contralateral knee or a sham control. Significance was determined by analysis of variance (ANOVA) with Tukey's post-hoc tests for multiple comparisons. Gene expression was normalised to an 18S internal control and the normalised expression for each gene is shown for the contralateral unoperated right knee, a sham control (left) knee and the operated left knee. Significance to the contralateral and sham controls is indicated on the top and bottom, respectively. **(b) **The expression of *Crabp2 *is still elevated 10 weeks post-surgery. Significance in gene expression at 10 weeks post-surgery was determined by two-tailed t-test analysis of the left (L, operated) versus right (R, contralateral control) knee. No sham was included in the 10-week group. * p < 0.05; ** p < 0.01; ns = not significant.

To quantify gene expression in single knee joints we developed efficient RNA isolation methods that yielded about 100 to 150 ng of total RNA from individual stifle joints sufficient for analysis of five to seven genes (20 ng of RNA per gene). Previous studies have shown that *Col1a1 *and *Col10a1 *are both elevated in OA [35-38]. We also examined the expression of *Sox9*, a transcription factor important in chondrocyte differentiation and matrix production [39]. With the exception of the four-week time point, the expression of *Col1a1 *was found to be elevated more than three-fold at all times in the MTX/PMX joint in comparison to the sham control (Figure 4). In contrast, there were only slight changes in the expression of *Col10a1 *or *Sox9 *during the first six weeks after surgery (Figure 4), with a significant (n = 5, p < 0.05) two-fold increase in *Col10a1 *being observed in six-week PMX samples in comparison to the sham control (Figure 4). Interestingly, examination of *Crabp2 *expression revealed a large increase in expression at two weeks after surgery in both MTX and PMX of about 18-fold and 10-fold, respectively (n = 5; MTX, p < 0.05; PMX, p < 0.01; Figure 5a). This magnitude of induction declined over time; however, by six weeks in the PMX mice there was about a three-fold increase in *Crabp2 *expression in PMX knees in comparison to the sham or contralateral knee (n = 5, p < 0.05; Figure 5a), whereas at 10 weeks, there was still about a five-fold increase in *Crabp2 *expression in PMX knees in comparison to the contralateral control (n = 7, p < 0.05; Figure 5b). Together these results show that PMX and MTX surgeries lead to a slowly progressing arthrosis in mice and that *Crabp2 *represents an early and sustained marker of DJD.

*Crabp2 *expression is regulated by retinoic acid and its increase in expression is consistent with activation of this pathway. CRABP2 has been shown to function both to suppress retinoic acid receptor activity by sequestering ligands and also as a vehicle to deliver ligands to the retinoic acid receptors, thereby enhancing ligand-mediated retinoic acid receptor transcriptional activity [40-45]. More recent reports favour the latter function, indicating that increased expression of *Crabp2 *enhances retinoic acid receptor transcriptional activity [41,42,44]. Further, *Crabp2 *null animals present with minor limb defects including an extra post-axial digit that (based on more recent reports) would suggest decreased retinoid signalling in the absence of CRABP2 [9,46,47]. In aggregate, increased expression of *Crabp2 *is generally linked to activation of the retinoid pathway either directly, because expression of *Crabp2 *is regulated by retinoic acid, or indirectly, because increased CRABP2 would increase retinoic acid receptor transcriptional activity. Inappropriate activation of the retinoic acid signalling pathway is expected to enhance cartilage degradation and/or chondrocyte dedifferentiation, both of which are observed in the osteoarthritic process.

As mentioned above, retinoic acid has been commonly used to promote degeneration in cartilage explants, and this has been associated with increased activity of various aggrecanases. Knockout animals of the gene encoding the aggrecanase ADAMTS5 are protected to a great extent from OA in joint instability models, indicating that this enzyme may play a major role in cartilage catabolism, at least in mouse models of OA [48-50]. Interestingly, in explants derived from double mutants of *Adamts5 *and another major aggrecanase *Adamts4*, addition of retinoic acid was still found to promote release of aggrecan through cleavage in the CS-2 domain [51]. Retinoic acid has been shown to increase *Adamts5 *and *Mmp13 *expression [19,22], and these new findings by Rogerson and colleagues [51] suggest that retinoic acid may also be promoting cartilage degradation through additional and as yet undefined aggrecanase(s). Further, in collagen-induced arthritis in the mouse and streptococcal cell wall-induced arthritis in rats, small molecule antagonists of the retinoic acid receptors were found to ameliorate pain and decrease cartilage loss [22].

In addition to *Crabp2*, other components of the retinoid signalling pathway were found to be significantly elevated in the joints of the aforementioned rat OA joint instability model, including genes encoding proteins involved in retinoic acid synthesis (*Aldh1a3*) and retinol transport and delivery (*Lrat *and retinol dehydrogenase) and a putative retinoic acid target gene (*Stra3*) [30]. Together, these findings along with our observations of elevated *Crabp2 *expression in mouse models of DJD suggest that retinoic acid may play a fundamental and perhaps unappreciated role in the osteoarthritic process.

As *Crabp2 *is robustly expressed in early OA, it may represent a marker for detection of early OA. Further, polymorphisms in *FRZB *and *GDF5 *have been linked to OA, and similar to the retinoid signalling pathway, they all play a role in endochondral ossification and modulation of their activity may impact maintenance of the articular chondrocyte [1]. In this regard, as has been previously suggested, antagonists of the retinoic acid signalling pathway may prove useful for maintaining the chondrocyte phenotype [8].

## Conclusion

The joint instability models presented herein in contrast to more aggressive models involving for instance ACL transection, present with OA-like pathology but still appreciable articular cartilage 10 weeks after surgery, thereby enabling the use of molecular approaches to quantify gene expression changes in early OA [34].

The expression of the retinoic acid-regulated gene *Crabp2 *is significantly elevated in early DJD, and may be a useful marker to follow early changes in cartilage in response to joint instability or in OA. Manipulation of the retinoic acid signalling pathway may prove useful in modifying the clinical course of OA.

## Abbreviations

ACAN: aggrecan; ACL: anterior cruciate ligament; ANOVA: analysis of variance; DMM: destabilisation of the medial meniscus; DJD: degenerative joint disease; ECM: extracellular matrix; MCL: medial collateral ligament; MTX: meniscotibial transection; OA: osteoarthritis; PMX: partial meniscoectomy; RT-qPCR: reverse transcription quantitative polymerase chain reaction.

## Competing interests

The authors declare that they have no competing interests.

## Authors' contributions

IW performed experiments, contributed to experimental design, writing of the manuscript and data interpretation. MC contributed to experimental design and carried out the experiments. FM and TMU were involved in experimental design, data interpretation and writing of the manuscript.
